# “Polymers from Renewable Resources”: Key Findings from This Topic Special Issue

**DOI:** 10.3390/polym15153300

**Published:** 2023-08-04

**Authors:** Valentina Siracusa, Nadia Lotti, Michelina Soccio, Alexey L. Iordanskii

**Affiliations:** 1 Department of Chemical Sciences, Università degli Studi di Catania, 95125 Catania, Italy; 2 Department of Civil, Chemical, Environmental and Materials Engineering of the University of Bologna, 40131 Bologna, Italy; nadia.lotti@unibo.it (N.L.); m.soccio@unibo.it (M.S.); 3 N. N. Semenov Federal Research Center for Chemical Physics, Russian Academy of Sciences, 119991 Moscow, Russia

## 1. Introduction

The Food and Agriculture Organization of the United Nations (FAO) has estimated that about one-third of the food produced for human consumption is currently lost or wasted, resulted in an estimated approximately USD 750 billion of direct costs for food producers every year. Of course, packaging plays a key role in reducing food waste and in food preservation and protection due to its innovative properties and eminent versatility of applications. Polymers and their composites used as packaging plastics have changed global needs and to the progress of modern technologies. However, plastic packaging is still considered in the framework of the outdated paradigm, “make, use, and dispose it”, with great material and energy losses incurred after the disposal of plastics. Consequently, hundreds of millions of tons of plastics are lost due to disposal practices and are discarded throughout the environment. The most interesting challenge for the future is the use of food waste and waste in general as a renewable resource to produce new bio-based and/or biodegradable materials with well-tailored properties. The circular economy is based on a new, important concept: eliminate waste, rebuild natural capital, and create economic value by using and not consuming resources. This game-changing strategy includes the promotion of sustainable polymer technologies that are able to exclude plastics from fossil resources, use renewable resources obtained from food or natural wastes as principal feedstocks, reduce the presence of plastic wastes in the environment, and increase the quality and uptake of recycling.

## 2. Scope of This Special Issue

The purpose of this special collection was to cover all the topics related to biomaterials obtained from renewable resources, including innovative feedstock, polymerization processes, full characterizations, final applications and life cycle assessment analyses in order to share all the academic and industrial efforts related to new and innovative sustainable materials and technologies. Nineteen papers were published in this Topic Special Issue: one in *Sustainability*, two in *Polysaccharides*, and sixteen in *Polymers*. A brief account of the research presented in the Special Issue is provided below.

## 3. Overview of the Papers Included in This Topic Special Issue

Ichim and collaborators published an article in *Sustainability* [1]. They investigated the properties of composite materials that contain 50% hemp fibers as a reinforcement and 50% recycled polypropylene (rPP) as a matrix (50/50 hemp/rPP). These were produced using thermoforming in collaboration with a Romanian furniture manufacturing company. The effect of the addition of a compatibilizer, maleic anhydride, was investigated. The aim was to understand how the waste recycling could reduce the environmental impact caused by waste landfilling or incineration. Composites incorporating 50% and 100% recycled fibers were treated with 2.5% and 5% maleated polypropylene (MAPP), respectively, and compared to both the untreated composites. The addition of recycled fibers decreased the mechanical performance, while the addition of just 5% of MAPP caused an improvement in the mechanical properties of the composites containing both 50 and 100% recycled fibers. The selected materials obtained by replacing wood with composite materials allow the manufacturing of high-quality sustainable products at a low price, with significant environmental benefits for climate regulation and pollution prevention, reducing the pressure on forests, and closing the loop in the circular economy.

Two articles were published in *Polysaccharides* by authors from Brazil. The first one [2] was focused on the synthesis and characterization of antimicrobial films based on methylcellulose/poly (vinyl alcohol) (MC/PVOH) incorporated with cellulose nanocrystals (CNC) and natamycin (MC/PVOH/CNC/natamycin blends films) for cheese preservation. The aim was to direct the attention to the possibility of guaranteeing a final product with a high microbiological quality, improving the product’s shelf life, minimizing the risks to consumers’ health, as well as reducing the economic losses due to food waste. To do this, new technologies were considered, such as the production of active packaging with the incorporation of antimicrobial substances into the polymer matrix of the packaging, thereby reducing the addition of preservatives directly into the food formulation. In order to improve the use of bioplastic, CNC nanostructures obtained from a renewable source (cellulose) were used. The mechanical and optical properties and avoidance of oxygen and water vapor permeation were evaluated, while the antimicrobial activity was tested against fungi and yeasts in vitro. Despite the incorporation of CNC, the films’ tensile strength was increased, and their addition did not influence the water vapor and oxygen barrier behavior. The incorporation of natamycin, an antimicrobial agent, had a negative effect on the optical and mechanical properties of the films, probably due to the lower compatibility among the antimicrobial and the polymers. The authors concluded that the films showed the potential to be applied in cheese preservation, thanks to their beneficial effect against fungi and yeasts, but more studies must be conducted in order to improve the mechanical, optical, and barrier properties of these active films.

The second paper [3] reports on the use of a totally green process based on reactive extrusion or the production of cassava starch hydrogels via a reaction with two organic crosslinking agents, citric (CA) and tartaric (TA) acids. Hydrogels are materials that can be produced using natural or synthetic polymers in different formats, including films, membranes, powders, and micro- or nanogels, with the ability of retain water or biological fluids without dissolving them. Starch is a biodegradable, non-toxic, and inexpensive biopolymer that is available worldwide. In order to obtain food-grade starch hydrogels, CA and TA non-toxic reagents were safely used in reactive extrusion, without using other reagents. As reported by the authors, reactive extrusion is a continuous process that has commercial viability; it is easy to adapt to industrial scales, offering a short reaction time (2–3 min). The physicochemical and microstructural properties of the obtained films confirmed the suitability of this green procedure in obtaining food-grade starch hydrogels with good performances, greatly reducing the processing time and effluent generation compared to those of conventional processes.

Sixteen other papers were published in *Polymers*. In particular:

- Six manuscripts described the results based on the modification of Polylactic acid (PLA), one of the most frequently used bio-based materials in the food packaging industry, which is applied for the production of disposable tableware, vegetables packaging, and fast food containers. Marano and collaborators [4] reported on the state-of-the-art barrier properties of poly(lactide) (PLA). Until now, many efforts have been made to meet precise functional requirements, such as suitable thermal, mechanical, and gas barrier properties, in order to guarantee the foods’ quality and safety during the whole food shelf-life. In particular, the authors focused their attention on reviewing relevant strategies to tailor the barrier properties of PLA-based materials, with the ultimate goal of providing a general guide for the design of PLA-based packaging materials with the desired mass transfer properties for water vapor, oxygen, and/or carbon dioxide. After the revision of 295 articles, the authors concluded that several strategies could be considered in order to tailor the final PLA barrier properties, such as crystallization control and co-polymerization. Azevedo and collaborators [5] reported their results regarding the process-induced changes in the morphologies of biodegradable polybutylene adipate terephthalate (PBAT) and polylactic acid (PLA) blends modified with four multifunctional chain-extending crosslinkers (CECLs). Investigations on the PBAT/PLA blends showed that the interfacial compatibility between PLA and PBAT is poor, but it can be improved using compatibilizers in order to optimize their processability and usage performance. The CECL introduction into the blend changed the thickness of the PLA fibrils, modified the interface adhesion, and altered the deformation behavior of the PBAT matrix from brittle to ductile, proving that CECLs react selectively with PBAT, PLA, and their interface. The most synergetic effect was obtained when 1,3-phenylenebisoxazoline was used as a crosslinker. Patel and collaborators [6] described the properties of extruded Poly(lactic acid)-poly(hydroxybutyrate) (PLA-PHB)-based nanocomposite films, with bio-based additives (CNCs and ChNCs) and a oligomer lactic acid (OLA) compatibilizer included in the films at a pilot scale. The aim was to identify suitable material formulations and nanocomposite production processes for film production at a larger scale for food packaging applications. As is known, for foods sensitive to oxidation, packages with low oxygen permeability are preferred. It is well known that the crystalline phase has a significant influence on the oxygen barrier properties of a material. As a consequence, increasing the crystallinity of PLA by blending PLA with other more crystalline biopolymers, such as poly(hydroxy alkanoates) (PHAs), for example, has subsequently received a lot of attention in the food packaging industry. The most common PHA is poly(hydroxybutyrate) (PHB). In addition, the synergic effect of better-dispersed ChNCs with the assistance of OLA resulted in increased crystallinity, and, thereby, an improvement in the oxygen barrier performance modified films as compared to that of neat PLA. Rogovina and collaborators [7] analyzed the hydrolysis resistance, biodegradation-in-soil, and ion sorption behavior of film binary polyester–chitosan composites, such as polylactide (PLA)–chitosan and poly(3-hydroxybutyrate) (PHB)–chitosan. Chitosan combined with biodegradable polyesters (PLA and PHB) has been studied as a novel functional material capable of performing in aqueous environments and soil. They found that PHB-chitosan composites are more stable during acid hydrolysis, they demonstrate better degradation in soil, and have a higher capacity for iron ions sorption than PLA-chitosan composites do. Singh and collaborators [8] studied the effects of Indian rosewood waste on the thermal and dry sliding wear properties of poly(lactic acid) (PLA) biocomposites. The inclusion of some natural fibers and sustainable biocarbon was reported to enhance the wear resistance of PLA-based biocomposites. The results demonstrated that the thermal stability of the PLA biocomposites increased with an increase in the wood waste loading, the wear of the biocomposites increased with an increase in the load and sliding velocity, and a wood waste content of 46.82% was the most dominant parameter for controlling the wear of the biocomposites. Alexeeva and collaborators [9] reported a result obtained regarding films made via the introduction of Glycero-(9,10-trioxolane)-trioleate (ozonide of oleic acid triglyceride, OTOA) into polylactic acid (PLA) films. The morphological, mechanical, thermal, and water absorption properties of PLA films after OTOA addition were studied to understand their suitability for packaging as well as biomedical applications. It was found that OTOA acts as a plasticizer and leads to an increase in PLA segmental mobility, which, in turn, contributes to changes in the thermodynamic and mechanical properties of PLA films. Eventually, the obtained results evidence that the morphological, thermodynamic, and mechanical properties of PLA + OTOA films could be controlled according to the OTOA content in the films.
- Two articles described the use and properties of wood treated in order to improve its mechanical behavior. In particular, Xu and collaborators [10] described a green process used to improve fast-growing wood’s durability via impregnation modification. Generally, impregnation with low-molecular-weight resins can improve the properties of wood, but these resins release free phenols and free aldehydes into the environment. In contrast, furfuryl alcohol (FA) is a green modification agent derived from pentose-rich agricultural residues and releases fewer volatile organic compounds or polycyclic aromatic hydrocarbons during the combustion and degradation of FA-modified wood. In this study, balsa wood was first immersed in an FA solution, followed by in situ polymerization to obtain furfurylated wood. The obtained samples were investigated in order to understand the mechanism of interaction (crosslinking) between FA and lignin. Esteves and collaborators [11] studied the properties of particleboards made from very young Paulownia trees from Portuguese plantations. Paulownia wood is easily air dried, without severe drying defects occurring. It is more resistant to fires than other fast-growing species are due to its high ignition temperature, high water content in the fire season, and large leaves. In this study, single-layer particleboards were made from 3-year-old Paulownia trees using different processing parameters and different board composition in order to determine the best properties, such as the mechanical properties and lower thermal conductivity, for use in dry environments. Its fast-growing rate would allow sustainable forest management to be achieved since the wood can be harvested sooner than traditional wood species can be.
- Eight articles described different materials and processes with environmentally friendly attributes. Sriprom and collaborators [12] worked on a novel, reinforced, recycled expanded polystyrene (r-EPS) foam/natural fiber composite obtained via a dissolution method. Coconut husk fiber (coir) and banana stem fiber (BSF) were used as the reinforcement materials in order to enhance the mechanical properties. Nazarov and collaborators [13] described the synthesis of different optically active polymers used as materials for dense enantioselective membranes, as well as chiral stationary phases for gas and liquid chromatography. In particular, chiral polymers were obtained from Norbornenes using renewable chemical feedstocks. As result, a series of high-molecular-weight polymers with good film-forming properties was successfully obtained. Yi and collaborators [14] described the use of two diol chain extenders, bis(2-hydroxyethyl) (1,3-pheny-lene-bis-(methylene)) dicarbamate (BDM) and bis(2-hydroxyethyl) (methylenebis(cyclohexane-4,1-diy-l)) dicarbamate (BDH), required to construct self-healing thermoplastic polyurethane elastomers (TPU). Self-healing polyurethane materials are widely applied in electronic skin, intelligent sensors, biomedical materials, and many other areas. BDM and BDH were successfully synthesized from inexpensive raw materials and incorporated into the polyurethane backbone, resulting in an innovative strategy to explore elastomers with good mechanical properties and an excellent self-healing ability. Chien and collaborators [15] studied the degradation ability of a Polybutylene succinate-co-adipate (PBSA) biodegradable polymer used for packaging and mulching. In this study, two elite fungal strains for PBSA degradation from farmlands, i.e., *Aspergillus fumigatus* L30 and *Aspergillus terreus* HC, were isolated. Additionally, the biodegradability of PBSA films buried in farmland soils was evaluated. Lehman and collaborators [16] studied the influence of non-rubber components on the properties of unvulcanized natural rubber. Natural rubber latex (NRL) is obtainable from rubber plants in the form of latex. It can be contaminated by micro-organisms because it contains various nutritious substances otherwise known as non-rubber components. For this study, the fresh natural latex from four different clones (RRIM600, RRIT251, PB235, and BPM24) was chosen. Kim and collaborators [17] described the use of a polyvinyl chloride (PVC)–polyethyleneimine (PEI) composite fiber (PEI-PVC) as a recoverable adsorbent for the removal of phosphorus from aqueous phases. The adsorptive removal of phosphorus from discharged effluents has been recognized as one of the most promising solutions in the prevention of eutrophication. In addition, the regenerated PEI-PVC maintained a phosphorus sorption capacity almost equal to that of the first use through the desorption process, and the PEI-PVC fiber did not elute any toxic chlorines into the solution during light irradiation. Chipón and collaborators [18] analyzed the effect of cellulose nanocrystals (CNCs) on the gelatinization of different starches (potato, wheat, and waxy maize) via the characterization of the rheological and thermal properties of starch–CNC blends. Starch and cellulose are the most widely distributed polymers in nature, with starch being found in the form of granules, which are energy reservoirs for plants and cellulose and are a part of the cell walls in plant tissues. Despite the effect of CNCs on the physical properties and functionality of different starches being described in the literature, studies describing the role of CNCs during the gelatinization of starch are scarce. Therefore, this work aimed to study the gelatinization of starches from different sources in the presence of CNCs produced from cotton cellulose pulp. Zhao and collaborators [19] worked on a very interesting project. They prepared a magnetic fly ash/polydimethylsiloxane (MFA@PDMS) sponge using simple dip-coating PDMS-containing ethanol in a magnetic fly ash aqueous suspension that solidified. The presence of the PDMS matrix made the sponge super-hydrophobic, with a significant lubricating oil absorption capacity; notably, it took only 10 min for the material to adsorb six times its own weight in n-hexane (oil phase). Considering the sizable interest in the environment, the authors reported that MFA@PDMS sponge demonstrated outstanding recyclability and stability since no decline in its absorption efficiency was observed after more than eight cycles. The preparation process was simple, and the resulting magnetic sponges were super-hydrophobic and super-lipophilic. Additionally, the magnetic properties of the material make it possible to separate oil–water mixtures using an external magnetic field. The tested sponge showed good mechanical stability, oil stability, and reusability.

## 4. Conclusions

All the research results published in this Special Issue reported innovative procedures used to obtain new materials based on the concept of “waste to waste”. Due to global industrialization, water, soil, and air pollution are among the most difficult challenges today. They are not only harmful to the natural ecosystem, but also have long-term adverse effects on human health and the economy.
